# Longitudinal changes in blood pressure and fasting plasma glucose among 5,398 primary care patients with concomitant hypertension and diabetes: An observational study and implications for community-based cardiovascular prevention

**DOI:** 10.3389/fcvm.2023.1120543

**Published:** 2023-04-03

**Authors:** Xiao Yu, Yu Ting Li, Hui Cheng, Sufen Zhu, Xiu-Jing Hu, Jia Ji Wang, Bedru H. Mohammed, Yao Jie Xie, Jose Hernandez, Hua-Feng Wu, Harry H. X. Wang

**Affiliations:** ^1^ School of Public Health, Sun Yat-Sen University, Guangzhou, China; ^2^ Zhongshan Ophthalmic Center, Sun Yat-Sen University, Guangzhou, China; ^3^ Nuffield Department of Primary Care Health Sciences, University of Oxford, Oxford, United Kingdom; ^4^ Zhejiang Provincial Center for Disease Control and Prevention, Hangzhou, China; ^5^ Centre for General Practice, The Seventh Affiliated Hospital, Southern Medical University, Foshan, China; ^6^ Guangdong-provincial Primary Healthcare Association, Guangzhou, China; ^7^ School of Public Health, LKS Faculty of Medicine, The University of Hong Kong, Pokfulam, Hong Kong SAR; ^8^ School of Nursing, The Hong Kong Polytechnic University, Hung Hom, Hong Kong SAR; ^9^ Faculty of Medicine and Health, EDU, Digital Education Holdings Ltd., Kalkara, Malta; ^10^ Green Templeton College, University of Oxford, Oxford, United Kingdom; ^11^ Shishan Community Health Centre of Nanhai, Foshan, China; ^12^ JC School of Public Health and Primary Care, Faculty of Medicine, The Chinese University of Hong Kong, Shatin, Hong Kong SAR; ^13^ Usher Institute, Deanery of Molecular, Genetic & Population Health Sciences, The University of Edinburgh, Edinburgh, United Kingdom

**Keywords:** long-term follow-up, blood pressure, fasting plasma glucose, hypertension, diabetes, routine primary care, cardiovascular prevention

## Abstract

**Aims:**

To assess longitudinal changes in blood pressure (BP) and fasting plasma glucose (FPG) in primary care patients with concomitant hypertension and type 2 diabetes mellitus (T2DM), and to explore factors associated with patients' inability to improve BP and FPG at follow-up.

**Methods:**

We constructed a closed cohort in the context of the national basic public health (BPH) service provision in an urbanised township in southern China. Primary care patients who had concomitant hypertension and T2DM were retrospectively followed up from 2016 to 2019. Data were retrieved electronically from the computerised BPH platform. Patient-level risk factors were explored using multivariable logistic regression analysis.

**Results:**

We included 5,398 patients (mean age 66 years; range 28.9 to 96.1 years). At baseline, almost half [48.3% (2,608/5,398)] of patients had uncontrolled BP or FPG. During follow-up, more than one-fourth [27.2% (1,467/5,398)] of patients had no improvement in both BP and FPG. Among all patients, we observed significant increases in systolic BP [2.31 mmHg, 95% confidence interval (CI): 2.04 to 2.59, *p *< 0.001], diastolic BP (0.73 mmHg, 0.54 to 0.92, *p *< 0.001), and FPG (0.12 mmol/l, 0.09 to 0.15, *p *< 0.001) at follow-up compared to baseline. In addition to changes in body mass index [adjusted odds ratio (aOR)=1.045, 1.003 to 1.089, *p *= 0.037], poor adherence to lifestyle advice (aOR = 1.548, 1.356 to 1.766, *p *< 0.001), and unwillingness to actively enrol in health-care plans managed by the family doctor team (aOR = 1.379, 1.128 to 1.685, *p *= 0.001) were factors associated with no improvement in BP and FPG at follow-up.

**Conclusion:**

A suboptimal control of BP and FPG remains an ongoing challenge to primary care patients with concomitant hypertension and T2DM in real-world community settings. Tailored actions aiming to improve patients' adherence to healthy lifestyles, expand the delivery of team-based care, and encourage weight control should be incorporated into routine healthcare planning for community-based cardiovascular prevention.

## Introduction

Cardiovascular disease (CVD) represents one of the major public health challenges to population health worldwide (1). International evidence suggests that exposures to risk factors such as high systolic blood pressure (BP), high fasting plasma glucose (FPG), and high body mass index (BMI) have increased steadily (2). A recent modelling study on a global scale demonstrated that individuals presented with underlying long-term conditions, e.g., diabetes and cardiovascular disease, are at increased risk of severe COVID-19 (3). Hypertension and diabetes often occur together as a common modality of multimorbidity, which has become increasingly popular in the ageing population (4–7). The presence of concomitant hypertension and diabetes increases the risk for CVD events and mortality, and thus has imposed significant economic burdens on individuals, their families, and the healthcare system (8).

A substantial body of international guidelines suggest that primary care is one of the most cost-effective strategies for reducing morbidity, disability, and premature mortality attributed to hypertension and diabetes (8–12). Given the rising epidemic of both conditions, China's health-care reform has invested in a nationwide provision of free-of-charge, basic public health (BPH) service package to strengthen equitable primary care (13–16). Meanwhile, an emerging service delivery model entitled “family doctor team” has been piloted stepwise in primary care practice since 2016 as part of the “Healthy China Action (2019–2030)” national imitative (17–19). The family doctor team is characterised by general practice (GP) physicians working with public health practitioners, nurses and, if available and suitable, pharmacists and social workers, within a multidisciplinary primary care team (20). People with hypertension, type 2 diabetes mellitus (T2DM), or those aged 65 years and older are encouraged to actively enrol in the GP physician-led, team-based care through health-care registration. The team aims to serve as the first point of entry into the healthcare system, whilst enhancing preventive care through annual check-ups and tailor-made lifestyle advice alongside health education to support patients' self-management and population-based cardiovascular prevention.

However, there remain substantial physician- and system-level barriers to the management of hypertension and its complications, given the poor availability of manpower and limited clinical capacity in low-resource primary care settings where multi-component complex interventions are less common (21–23). The barriers are likely exacerbated by the traditional single disease approach (24, 25), coupled with “clinical inertia”, i.e., a common failure of physicians to initiate or intensify care regime when indicated (26), thus leading to difficulties in maintaining satisfactory control of BP and FPG over time (27, 28), with increased incidence of cardiovascular events (29). The hypothesis that multidisciplinary team-based care may overcome “clinical inertia” in the real-world community setting needs to be further tested. From a multimorbidity perspective, current knowledge on whether adherence to lifestyle advice and/or an active enrolment in routine health-care plans managed by the family doctor team may enhance patients' ability to achieve long-term improvement of BP and FPG in hypertensive patients with coexisting diabetes remains largely scant.

In this study, we aimed to assess the longitudinal changes in BP and FPG in Chinese primary care patients with concomitant hypertension and T2DM, and to explore independent factors associated with patients' inability to improve BP and FPG at follow-up.

## Methods

### Study design and data source

We constructed a closed cohort of primary care patients who attended free-of-charge, annual check-ups in the context of the national basic public health (BPH) service provision in an urbanised township consisting of 47 communities in southern China. The annual check-ups were performed onsite at community health centres (CHCs). The BPH service information platform has become routinely operational since 2016. At each check-up, public health staff at CHCs documented individuals' health reports electronically on the information platform. In this study, computerised data were retrospectively captured between 2016 and 2019.

### Participants

**Figure 1:**
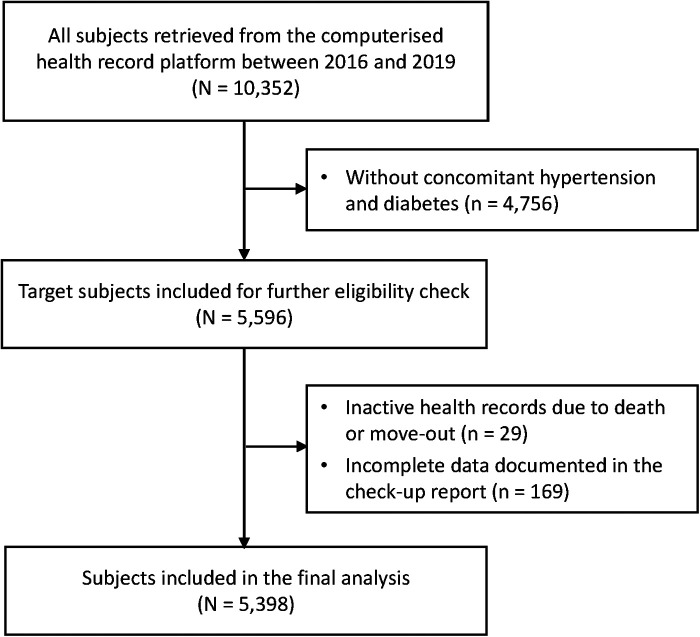
Diagram of study flow.

The target participants were adult primary care service users with concomitant hypertension and T2DM who attended annual check-ups at CHCs. Patients' earliest check-up attendance during the study period was regarded as a baseline and their most recent attendance in 2019 was considered a follow-up. Data from patients who had the coexistence of physician-diagnosed hypertension and T2DM were retrieved. Hypertension was considered present if an individual had systolic BP ≥ 140 mmHg and/or diastolic BP ≥ 90 mmHg on repeated clinical measurements, or had antihypertensive medication. Diabetes was defined as FPG ≥ 7.0 mmol/L, or on glucose-lowering therapies. Patients whose health records were inactive due to death or move-out and those with incomplete socio-demographic information were excluded from the analysis (Figure 1).

### Study variables and measurements

A data management checklist of variables needed for data analysis was jointly reviewed by a research panel consisting of two public health specialists (HHXW and YTL), two medical practitioners (XJH and HFW), and one epidemiologist (JH). The main outcome variables were systolic BP, diastolic BP, and FPG. The clinical measurement of BP was conducted in a seated position by routinely-validated automatic sphygmomanometers. The arm with the higher pressure was used. The average of two BP readings, 1–2 min apart, was recorded. A venous blood sample at fasting was collected on-site. FPG was determined by enzymatic methods according to standard operating procedures. All clinical measurements and laboratory tests had internal quality control in accordance with the national standard. The achievement of controlled BP and FPG at baseline was defined according to guidelines that advocate treating hypertension in people with diabetes to a BP goal <130/80 mmHg, along with FPG <7.0 mmol/L (10–12, 28, 30, 31). Age, sex, educational attainment, duration of follow-up, number of chronic diseases, number of antihypertensive and glucose-lowering medications taken, anthropometric parameters, adherence to lifestyle advice, and active enrolment in team-based care were patient-level independent variables captured in the study.

Weight was measured with light clothing and without shoes by a calibrated weighing scale, and height was measured using a wall-mounted stadiometer. The BMI was calculated as weight in kilograms divided by squared height in meters (kg/m^2^). Chronic diseases documented in the health records included hypertension, diabetes, coronary heart disease, stroke, chronic obstructive pulmonary disease, cancer, mental disorders, tuberculosis, and chronic kidney disease. Adherence to lifestyle advice was determined based on the presence of at least one of the following self-reported habits on a regular basis, i.e., salt consumption of <6 g per day, no smoking, moderate-intensity aerobic exercise for ≥180 min per week, and restricted daily alcohol intake (≤25 g for males or ≤15 g for females) (13, 30). Patients who had no responsible physician documented in the health record or who did not attend all consecutive annual check-ups (e.g., lipid profile test measuring the amount of cholesterol and triglycerides, body constitution assessment, and medication review if applicable, etc.) for a comprehensive set of health assessments since baseline registration were not deemed active enrolment in health-care plans managed by the family doctor team. The duration of follow-up was calculated as the time period between the earliest check-up attendance and the most recent check-up attendance in 2019 for each patient.

### Statistical analysis

Descriptive analysis was conducted to describe the basic information of study participants according to control of BP and FPG at baseline, and by improvement of BP and FPG at follow-up, respectively. The paired *t* test was used to compare the changes in BP and FPG between baseline and follow-up within each subgroup. The independent two-sample *t* test was used to compare within-group changes for patients with and without improved outcomes at follow-up. A patient's inability to improve BP and FPG was defined as having no reduction in both outcomes at follow-up compared to baseline. Multivariable logistic regression models were constructed in a backward stepwise approach to explore all independent predictor variables that were significantly associated with no improvement in both BP and FPG at follow-up. The baseline BP and FPG were fitted as covariates. We also assessed the regression model accuracy metrics, i.e., Akaike information criterion (AIC) and Bayesian information criterion (BIC). The incremental value of risk factors identified in the regression model for predicting no improvement in BP and FPG was explored by applying C-statistic, net reclassification index (NRI), and integrated discrimination index (IDI) (32). The predicted probability of no improvement in health outcomes was calculated using the marginal standardisation approach, which was considered appropriate for making inferences on the overall source population where the study sample was drawn (33). We also performed sensitivity analysis on top of the fitted regression models to visualise the extent to which the association of adherence to lifestyle advice or active enrolment in multidisciplinary team-based care with BP and FPG improvements may vary across subgroups with different BP and FPG levels at baseline. Data analyses were performed using Stata (version 15.1, StataCorp, TX) and R (version 4.1.1, Core Team, Vienna). A *p* value < 0.05 was considered statistically significant.

### Ethics consideration

Data anonymisation was achieved by removing all patient identifiers from the dataset prior to data analysis. Ethics approval was initially granted and subsequently renewed by the School of Public Health Biomedical Research Ethics Review Committee at Sun Yat-Sen University (Refs: SPH2016027 and SPH2019032) following the Declaration of Helsinki 2013.

## Results

### Characteristics of study participants

A total of 5,398 primary care patients who fulfilled the eligibility criteria were included in the final analysis (Figure 1). Patients with uncontrolled BP or FPG at baseline were older (i.e., aged 60 years and above), had a longer duration of follow-up, and had a greater number of medications taken compared to their counterparts with controlled BP and FPG at baseline (Table 1). At follow-up, slightly over half [54.3% (797/1,467)] of patients who had no improvement in BP and FPG were able to adhere to lifestyle advice, which were less than that [64.1% (2,518/3,931)] of patients with improved clinical parameters. The proportion of patients actively enrolled in GP-led team-based care was also significantly lower in the non-improved subgroup than in their counterparts (Table 2).

**Table 1:** Characteristics of study participants according to control of blood pressure and fasting plasma glucose at baseline.

Variables	Overall (*N *= 5,398)	Uncontrolled BP or FPG (*n *= 2,608)	Controlled BP and FPG (*n *= 2,790)	*P* value
Age, *n* (%)				<0.001
< 60 years	1,330 (24.6)	552 (21.2)	778 (27.9)	
60–70 years	2,215 (41.0)	1,135 (43.5)	1,080 (38.7)	
> 70 years	1,853 (34.4)	921 (35.3)	932 (33.4)	
Sex, *n* (%)				0.013
Male	2,324 (43.1)	1,077 (41.3)	1,247 (44.7)	
Female	3,074 (56.9)	1,531 (58.7)	1,543 (55.3)	
Education level, *n* (%)				<0.001
Below primary school	419 (7.8)	204 (7.8)	215 (7.7)	
Primary school	3,357 (62.2)	1,727 (66.2)	1,630 (58.4)	
Secondary school and above	1,622 (30.0)	677 (26.0)	945 (33.9)	
Months of follow-up, mean (SD)	22.4 (10.9)	29.0 (4.5)	16.3 (11.5)	<0.001
Number of chronic diseases, mean (SD)	2.16 (0.40)	2.17 (0.41)	2.15 (0.39)	0.089
Number of medications taken, mean (SD)	1.62 (0.78)	1.66 (0.76)	1.57 (0.79)	<0.001
Adherence to lifestyle advice, n (%)				0.002
No	2,083 (38.6)	1,063 (40.8)	1,020 (36.6)	
Yes	3,315 (61.4)	1,545 (59.2)	1,770 (63.4)	
Enrolment in team-based care, *n* (%)				<0.001
Non-active	4,547 (84.2)	1,992 (76.4)	2,555 (91.6)	
Active	851 (15.8)	616 (23.6)	235 (8.4)	
BMI at baseline (kg/m^2^), mean (SD)	24.68 (3.26)	24.61 (3.13)	24.73 (3.37)	0.177
Systolic BP at baseline (mmHg), mean (SD)	128.45 (6.59)	130.76 (7.72)	126.29 (4.31)	<0.001
Diastolic BP at baseline (mmHg), mean (SD)	78.28 (4.57)	80.10 (5.06)	76.58 (3.23)	<0.001
FPG at baseline (mmol/L), mean (SD)	6.02 (0.75)	6.08 (1.02)	5.97 (0.32)	<0.001

BMI, body mass index; BP, blood pressure; FPG, fasting plasma glucose; SD, standard deviation.

Data were presented as n (%) or mean (SD) where appropriate.

**Table 2:** Characteristics of study participants according to the improvement of blood pressure and fasting plasma glucose at follow-up.

Variables	Overall (*N *= 5,398)	BP and FPG not improved at follow-up (*n *= 1,467)	BP or FPG improved at follow-up (*n *= 3,931)	*P* value
Age, *n* (%)				0.481
< 60 years	1,330 (24.6)	378 (25.8)	952 (24.2)	
60–70 years	2,215 (41.0)	597 (40.7)	1,618 (41.2)	
> 70 years	1,853 (34.4)	492 (33.5)	1,361 (34.6)	
Sex, *n* (%)				0.056
Male	2,324 (43.1)	663 (45.2)	1,661 (42.3)	
Female	3,074 (56.9)	804 (54.8)	2,270 (57.7)	
Education, *n* (%)				0.264
Below primary school	419 (7.8)	102 (7.0)	317 (8.1)	
Primary school	3,357 (62.2)	907 (61.8)	2,450 (62.3)	
Secondary school and above	1,622 (30.0)	458 (31.2)	1,164 (29.6)	
Months of follow-up, mean (SD)	22.4 (10.9)	21.8 (11.1)	22.7 (10.8)	0.008
Number of chronic diseases, mean (SD)	2.16 (0.40)	2.17 (0.41)	2.15 (0.39)	0.118
Number of medications taken, mean (SD)	1.62 (0.78)	1.63 (0.79)	1.61 (0.77)	0.330
Adherence to lifestyle advice, *n* (%)				<0.001
No	2,083 (38.6)	670 (45.7)	1,413 (35.9)	
Yes	3,315 (61.4)	797 (54.3)	2,518 (64.1)	
Enrolment in team-based care, *n* (%)				<0.001
Non-active	4,547 (84.2)	1,280 (87.3)	3,267 (83.1)	
Active	851 (15.8)	187 (12.7)	664 (16.9)	
BMI at follow-up (kg/m^2^), mean (SD)	24.84 (3.11)	25.15 (3.26)	24.73 (3.05)	<0.001
Systolic BP at follow-up (mmHg), mean (SD)	130.76 (8.78)	135.56 (7.98)	128.97 (8.38)	<0.001
Diastolic BP at follow-up (mmHg), mean (SD)	79.00 (5.74)	82.50 (4.86)	77.70 (5.50)	<0.001
FPG at follow-up (mmol/L), mean (SD)	6.14 (1.01)	6.64 (1.14)	5.96 (0.88)	<0.001

BMI, body mass index; BP, blood pressure; FPG, fasting plasma glucose; SD, standard deviation.

Data were presented as n (%) or mean (SD) where appropriate.

### Changes in BP and FPG between baseline and follow-up

Among all participants, systolic BP (2.31 mmHg, 95%CI: 2.04 to 2.59 mmHg, *p *< 0.001), diastolic BP (0.73 mmHg, 0.54 to 0.92 mmHg, *p *< 0.001), and FPG (0.12 mmol/L, 0.09 to 0.15 mmol/L, *p *< 0.001) increased consistently between baseline and follow-up. Compared to participants who had improved clinical parameters at follow-up, the between-group net changes in systolic BP, diastolic BP, and FPG were 9.22 mmHg (8.65 to 9.79 mmHg), 7.20 mmHg (6.82 to 7.58 mmHg), and 0.99 mmol/L (0.92 to 1.06 mmol/L), respectively, at follow-up (Table 3).

**Table 3:** Changes in blood pressure and fasting plasma glucose between baseline and follow-up.

Variables	Overall changes (95%CI)	Within-group changes		Between-group net changes (95%CI)
		BP and FPG not improved at follow-up	BP or FPG improved at follow-up	
Systolic BP (mmHg)	2.31 (2.04 to 2.59)^a^	9.03 (8.62 to 9.43)^a^	−0.19 (–0.51 to 0.13)	9.22 (8.65 to 9.79)^a^
Diastolic BP (mmHg)	0.73 (0.54 to 0.92)^a^	5.97 (5.72 to 6.23)^a^	–1.23 (–1.43 to –1.02)^a^	7.20 (6.82 to 7.58)^a^
FPG (mmol/L)	0.12 (0.09 to 0.15)^a^	0.84 (0.78 to 0.90)^a^	–0.15 (–0.18 to –0.11)^a^	0.99 (0.92 to 1.06)^a^

BP, blood pressure; FPG, fasting plasma glucose; SD, standard deviation; CI, confidence interval.

^a^ Within-group changes with *P* value less than 0.001. The paired *t* test was used to compare the changes in blood pressure and fasting plasma glucose between baseline and follow-up within each subgroup. The independent two-sample *t* test was used to compare the within-group changes for patients with and without improved outcomes at follow-up.

### Factors associated with no improvement in BP and FPG at follow-up

**Figure 2:**
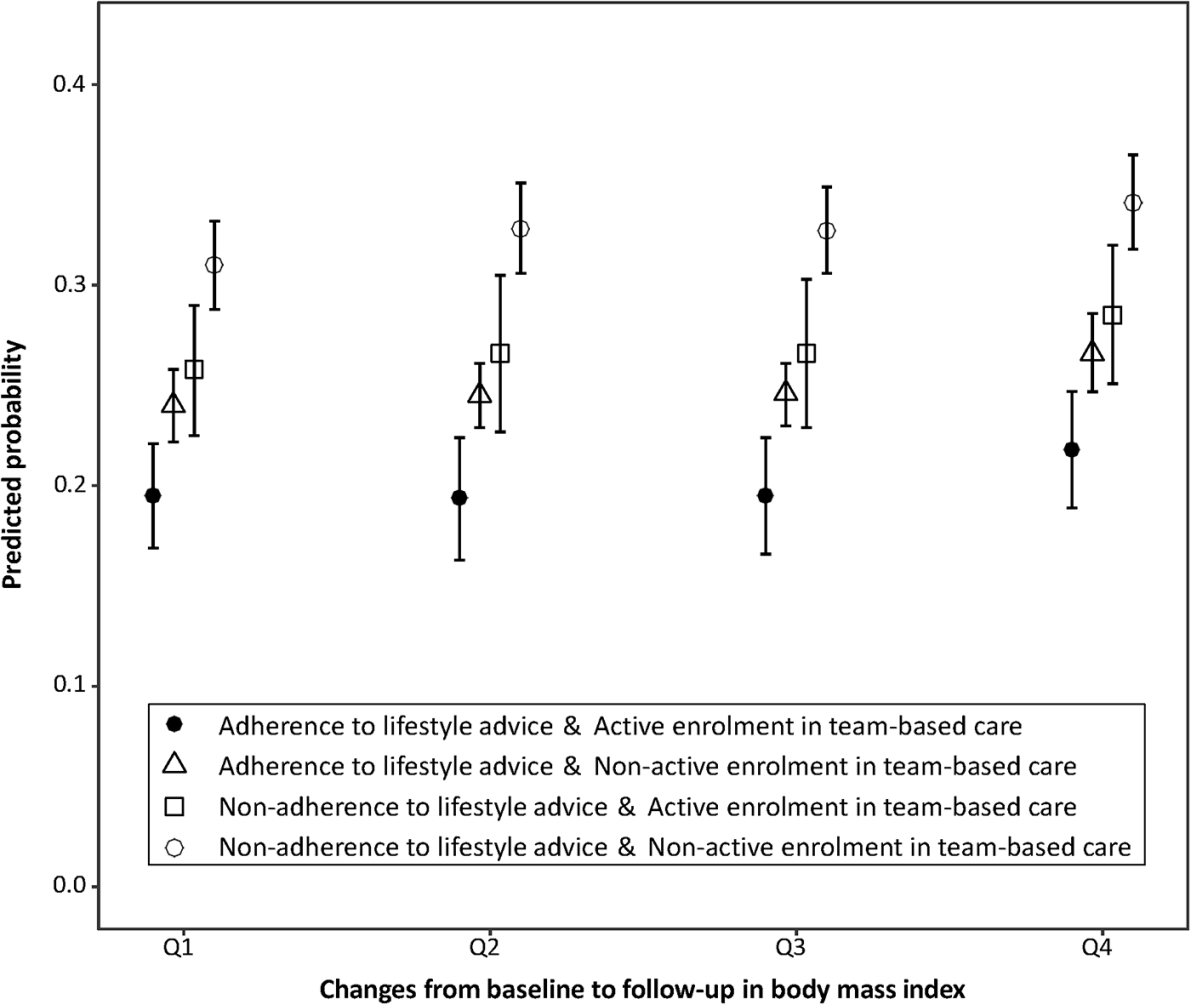
Predicted probability of no improvement in BP and FPG at follow-up. Note: Changes from baseline to follow-up in body mass index were divided into quartiles from greatest weight gain (Quartile 4; median change: 1.46 kg/m^2^) to least gain or loss (Quartile 1; median change: −1.00 kg/m^2^).

Multiple regression analysis revealed that non-adherence to lifestyle advice (aOR = 1.548, 95%CI: 1.356 to 1.766, *p *< 0.001), non-active enrolment in health-care plans managed by the family doctor team (aOR = 1.379, 1.128 to 1.685, *p *= 0.001), and increases in BMI during follow-up (aOR = 1.045, 1.003 to 1.089, *p *= 0.037) were independently associated with no improvement in BP and FPG after adjusting for confounding (Table 4). The estimates of NRI and IDI suggested that the predictive accuracy significantly increased by adding the three risk factors identified for the study outcome (Supplementary Table S1). We further conducted subgroup analysis to explore the predicted probability of no improvement in BP and FPG based on fitted multivariable regression models. When broken down by quartiles of changes in BMI from baseline to follow-up, we saw consistent associations between non-adherence to lifestyle advice or non-active enrolment in team-based care and no improvement in BP and FPG, accompanied by a synergistic effect. Compared with patients who had the least weight gain, adhered to lifestyle advice, and were actively enrolled in team-based care, those who had the greatest weight gain, failed to adhere to lifestyle advice, and were not actively enrolled in health-care plans managed by the family doctor team were most likely to have no improvement in BP and FPG at follow-up (Figure 2). Results were consistently visualised across different subgroups according to patients' BP and FPG levels at baseline, suggesting the robustness of the study findings (Supplementary Figure S1).

**Table 4:** Factors associated with no improvement in blood pressure and fasting plasma glucose at follow-up.

	Model 1		Model 2		Model 3	
	cOR (95%CI)	*P*	aOR (95%CI)	*P*	aOR (95%CI)	*P*
Adherence to lifestyle advice						
Yes	1.000 (Reference)		1.000 (Reference)		1.000 (Reference)	
No	1.498 (1.326–1.692)	<0.001	1.469 (1.299–1.661)	<0.001	1.548 (1.356–1.766)	<0.001
Enrolment in team-based care						
Active	1.000 (Reference)		1.000 (Reference)		1.000 (Reference)	
Non-active	1.391 (1.168–1.657)	<0.001	1.391 (1.164–1.662)	<0.001	1.379 (1.128–1.685)	0.001
Changes in BMI	1.029 (0.993–1.066)	0.115	1.027 (1.008–1.046)	0.004	1.045 (1.003–1.089)	0.037
Number of medications taken	1.039 (0.962–1.122)	0.330	1.037 (0.960–1.121)	0.358		
Number of chronic diseases	1.124 (0.970–1.303)	0.119	1.088 (0.936–1.264)	0.273		
Age						
< 60 years	1.000 (Reference)		1.000 (Reference)			
60–70 years	0.929 (0.798–1.081)	0.343	0.959 (0.816–1.128)	0.616		
> 70 years	0.910 (0.778–1.066)	0.243	0.982 (0.823–1.170)	0.835		
Sex						
Male	1.000 (Reference)		1.000 (Reference)			
Female	0.887 (0.786–1.001)	0.052	0.933 (0.823–1.059)	0.283		
Education						
Below primary school	1.000 (Reference)		1.000 (Reference)			
Primary school	1.151 (0.909–1.456)	0.244	1.115 (0.876–1.419)	0.378		
Secondary school and above	1.223 (0.954–1.567)	0.112	1.149 (0.879–1.503)	0.309		
Months of follow-up						
< 12 months	1.000 (Reference)		1.000 (Reference)			
≥ 12 months	0.987 (0.832–1.171)	0.879	1.038 (0.870–1.238)	0.680		

Note: cOR, crude odds ratio; aOR, adjusted odds ratio; CI, confidence interval; BMI, body mass index.

Dependent variable: no improvement in both blood pressure and fasting plasma glucose (*Y* = 1) *vs.* either improvement in blood pressure or fasting plasma glucose (*Y* = 0). All independent predictor variables that were statistically significant in the saturated model (Model 2) were included in the final model (Model 3). The baseline BP and FPG were fitted as covariates.

## Discussion

### Main findings

In a community-based, longitudinal cohort of Chinese primary care patients with concomitant hypertension and T2DM who were followed for a mean of 22.4 months, we found that more than one-fourth of patients had no improvement in both BP and FPG. Greater increases in BMI, poor adherence to lifestyle advice, and unwillingness to actively enrol in team-based care were factors independently associated with no improvement in BP and FPG over time.

### Relationship with other studies

Hypertension and T2DM are among the most prevalent chronic conditions worldwide. The two conditions often occur together, which not only complicate treatment strategy and increase healthcare costs, but also heightens the risk for CVDs considerably (4). A recent study suggested that people at high risk for either hypertension or diabetes share common risk factors including abdominal obesity, hyperinsulinemia, and hypertriglyceridemia, and that weight gain may contribute to the development of both hypertension and diabetes mellitus (34). Although lowering BP and blood glucose has been recommended in the prevention of CVDs, control of BP and blood glucose in real-world settings is often unsatisfactory (35, 36). Consistent with our results, a cross-sectional, population-based study showed that fewer than half of the participants on medication for hypertension or diabetes had adequately controlled BP or FPG, suggesting the unmet need for effective health system intervention to improve access to care (37). Evidence from community-based, randomised controlled trials conducted in resource-rich settings such as the UK and Hong Kong showed that “real-world” prevention programmes may not always lead to successful reduction in BP, blood glucose, and other cardiovascular risk factors (38, 39). Hence, numerous efforts are still needed to translate trials from research-based settings that are effective to daily practice where intensive efforts at CVD risk reduction are less likely to be sustained regularly due to the possible co-existence of clinical inertia and physician burnout (40, 41).

Consistent with previous findings from the mendelian randomisation study and cross-sectional investigation that suggested a strong association between BMI and cardiometabolic disease (42, 43), longitudinal analysis of data in our cohort showed that patients who had their BMI levels increased during the follow-up tended to have no improvement in BP and FPG, implying the need for maximising the patient's motivation to maintain continuous monitoring of weight gain and engage in regular aerobic exercises that are necessary to primary prevention of CVD (44). A most recent review discussed the main physiological mechanisms that underpinned the beneficial effects of optimal lifestyles on BP control and overall cardiovascular health, advocating the use of lifestyle interventions for the prevention and adjuvant treatment of hypertension (45). Despite solid evidence on the association between lifestyles and cardiovascular-related clinical outcomes (46), the rising epidemic of unhealthy lifestyles remains a major challenge to the traditional medical practice model (47).

Our findings provided evidence to fill in the gaps in understanding of whether multidisciplinary team-based primary care may overcome “clinical inertia” in the real-world setting. International experiences from Canada and Israel demonstrated that inter-professional teams could contribute to facilitating the transfer of knowledge, skills and attitudes, and thus are capable of enhancing clinical competencies and overcoming traditional barriers in the delivery of cardiovascular care (48–50). Alongside the transformation of practice paradigm to empower hypertensive patients with the coexistence of common long-term conditions such as T2DM, team-based educational programmes and tools that accommodate the needs of and provide support for underserved subpopulations might create opportunities to deepen patients' insights into the clustering of disease components and improve their intrinsic motivation to build self-management skills for cardiovascular health (51, 52).

### Strengths and weaknesses of the study

We constructed a primary care cohort of a large sample of hypertensive patients with coexisting diabetes whose data were retrieved from a computerised system, where healthcare record was documented according to standard procedures which ensured the accuracy of data. Objective measures were used to assess the longitudinal changes of BP and FPG to avoid subjective bias. The analysis was conducted from a multimorbidity perspective and the non-improvement in BP and FPG were considered as a combined outcome to take into account the joint or synergistic effect of CVD risk factors. However, there are several limitations of this study. First, we did not adopt a trial design where a “pure” control group is included, given that the main purpose of the present study was not to evaluate the effectiveness of either a particular lifestyle advice or a well-designed health-care plan managed by the family doctor team *per se*. Instead, we are interested in assessing factors associated with poor control of clinical parameters, i.e., BP and FPG, that are commonly seen in the context that real-world, routine primary care is delivered. Second, the study population was drawn from an urbanised township, which may limit the application of our findings to other populations in more socio-economically developed regions. Third, self-report lifestyle habits may be subject to recall bias, although adherence to lifestyle advice was conceptualised as a composite variable to minimise the extent to which the actual situation may deviate from single measures. Last but not least, confounding factors such as income, family history, dietary intake, medication adherence, and sleep history may also play a role but were not captured in the study due to the absence or inconsistent measurements of these variables in the original healthcare record across different years.

### Implications for research and clinical practice

Our findings that most of hypertensive patients with coexisting T2DM have experienced no improvements in BP and FPG over time indicated the necessity of continuous efforts to engage patients as active participants, rather than passive recipients, and to deliver effective care that is sustainable and accountable to individuals at elevated cardiovascular risk. The association between an active enrolment in team-based care and a higher probability of improved BP and FPG levels may reflect the patient-centred approach and a public health perspective adopted by the family doctor team in their daily work across the continuum of care. Despite the debate about the target BP that should be attained in diabetic patients, a rich body of evidence has confirmed the benefits of BP reduction alongside glycaemic control (53, 54). In light of a rapid increase in the number of people living with hypertension and T2DM, strategies to target subjects who may be less likely to improve neither BP nor FPG over time shall be crucial in the research agenda. Our analysis further showed that those who were more likely to have no improvement in BP and FPG over time tended to have greater weight gain. This may reflect the complexity of managing hypertension and its comorbidities that require patients' excessive time and intrinsic motivation to tackle a variety of treatment workloads including doctor visits, self-monitoring, and lifestyle changes. In this regard, counselling skills that enable primary care practitioners to have effective communication with patients may be of paramount importance (55). This would help enhance patient engagement in team-based care and improve shared-decision making in the long-term behavioural changes to achieve the national and international goals in CVD prevention and control.

## Conclusion

Suboptimal control of BP and FPG remains an ongoing challenge to primary care patients with concomitant hypertension and T2DM in real-world community settings. Tailored actions aiming to improve patients' adherence to healthy lifestyles, expand the delivery of family doctor team-based care, and encourage weight control should be incorporated into routine healthcare planning for community-based cardiovascular prevention.

## Data Availability

The raw data supporting the conclusions of this article are available on reasonable request from the corresponding author.
