# Supplementary material for: Longitudinal changes in blood pressure and fasting plasma glucose among 5,398 primary care patients with concomitant hypertension and diabetes: An observational study and implications for community-based cardiovascular prevention
Source: Front Cardiovasc Med. 2023 Apr 3;10:1120543. doi: 10.3389/fcvm.2023.1120543 (PMC10106827; doi:10.3389/fcvm.2023.1120543)
Supplement: Supplementary file 1 [file Datasheet1.pdf]

**Supplementary Figure S1 Predicted probability of no improvement in BP and FPG at follow-up: sensitivity analysis by subgroups**

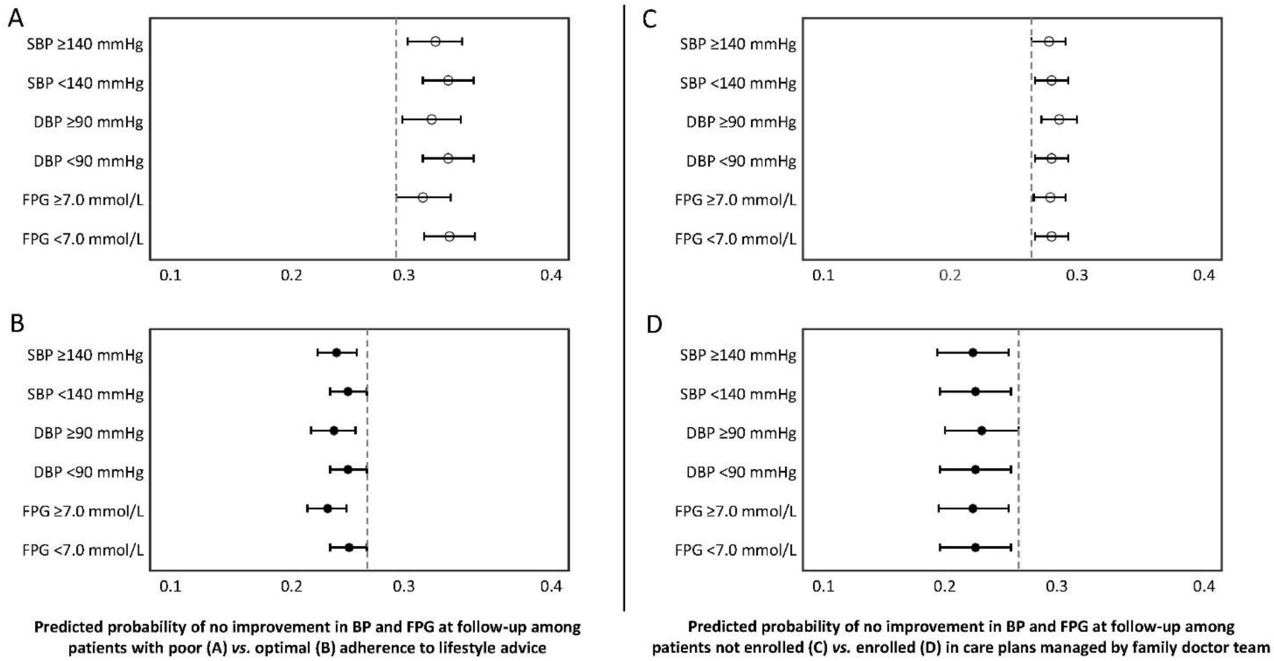


Note: SBP, systolic blood pressure; DBP, diastolic blood pressure; FPG, fasting plasma glucose.

Predicted probability of no improvement in BP and FPG at follow-up among by adherence to lifestyle advice (A: poor adherence *vs.* B: optimal adherence) and by enrolment in care plans managed by family doctor team (C: not actively enrolled *vs.* D: actively enrolled). Patients were divided into 6 different subgroups according to their BP and FPG levels at baseline. Error bars indicate 95% confidence intervals.

**Supplementary Table S1 Improvement in risk prediction model after adding changes in body mass index, adherence to lifestyle advice, and active enrolment in team-based care**

| | **C-statistic** | | **IDI** | | **NRI (categorical)** | | **NRI (continuous)** | |
|---|---|---|---|---|---|---|---|---|
| | Estimate (95%CI) | *P* | Estimate (95%CI) | *P* | Estimate (95%CI) | *P* | Estimate (95%CI) | *P* |
| Basic model | 0.724 (0.709-0.739) | | Reference | | Reference | | Reference | |
| Final model | 0.733 (0.718-0.748) | 0.337 | 0.010 (0.007-0.013) | <0.001 | 0.032 (0.004-0.061) | 0.026 | 0.211 (0.152-0.270) | <0.001 |

Note: IDI, integrated discrimination improvement; NRI, net reclassification index; CI, confidence interval. The basic model included age, sex, educational attainment, duration of follow-up, number of chronic diseases, number of antihypertensive and glucose-lowering medications taken, and baseline covariates. The final model was further fitted with changes in body mass index, adherence to lifestyle advice, and active enrolment in team-based care.
